# Aerobic Exercise Combined with Multisensory Stimulation Training Improves Cognitive Frailty by Modulating Circulating Klotho

**DOI:** 10.3390/ijms27093991

**Published:** 2026-04-29

**Authors:** Minguang Yang, Yuwei Ye, Liumu Wang, Dongrui Chi, Zhongyi Hu, Huawei Lin, Liming Chen, Yuxi Qiu, Yaling Dai, Jianhong Li, Weilin Liu, Jing Tao, Lidian Chen

**Affiliations:** 1 National-Local Joint Engineering Research Center of Rehabilitation Medicine Technology, Fujian University of Traditional Chinese Medicine, Fuzhou 350122, China; nick123321@126.com (M.Y.); yuweiye1995@163.com (Y.Y.); liuweilin12@fjtcm.edu.cn (W.L.); 2 The Institute of Rehabilitation Industry, Fujian University of Traditional Chinese Medicine, Fuzhou 350122, China; 3 College of Rehabilitation Medicine, Fujian University of Traditional Chinese Medicine, Fuzhou 350122, China; liumuw1996@outlook.com (L.W.); 1147897988@163.com (D.C.); h2405941296@163.com (Z.H.); lhw1433312752@163.com (H.L.); chenliming5165@outlook.com (L.C.); qiuyuxi1020@163.com (Y.Q.); yalingdai673@gmail.com (Y.D.); lijianhong25@fjtcm.edu.cn (J.L.)

**Keywords:** cognitive frailty, hippocampus, skeletal muscle, Klotho, FGFR1

## Abstract

Cognitive frailty (CF), characterized by concurrent cognitive and motor decline, is a major challenge to healthy aging, yet effective interventions remain limited. Klotho, an anti-aging protein that declines with age, has been implicated in both hippocampal function and skeletal muscle homeostasis. In this study, we investigated whether aerobic exercise combined with multisensory stimulation training (CT) ameliorates age-related CF through systemic Klotho signaling. A 16-month-old mouse model of age-related CF was assigned to aerobic training, multisensory stimulation, or combined training, and behavioral, electrophysiological, histological, and molecular assessments were performed. To examine the mechanistic role of Klotho, dual-route shRNA delivery was used to inhibit systemic Klotho expression. CT significantly improved cognitive and motor performance compared with either intervention alone. CT also increased hippocampal dendritic spine density and long-term potentiation, reduced collagen deposition in gastrocnemius muscle, and upregulated Klotho, FGF19, and FGFR1 expression in both hippocampus and muscle, accompanied by elevated serum Klotho levels. Klotho knockdown attenuated these beneficial effects, reduced PSD95 and GluN2B expression, and increased MuRF3 and TNF-α levels. These findings suggest that CT alleviates cognitive frailty and that systemic Klotho is a key mediator linking hippocampal synaptic function and skeletal muscle homeostasis.

## 1. Introduction

With the increase in the global elderly population, the prevalence of aging-related diseases is continuously rising [1]. In 2013, the International Academy of Nutrition and Aging (IANA) and the International Association of Gerontology and Geriatrics (IAGG) first proposed the concept of Cognitive Frailty (CF) [2], characterized by the concurrent decline in cognitive and motor functions. This condition significantly impairs quality of life and increases mortality risk, emerging as a major threat to healthy aging [3,4].

The biological factors contributing to CF remain unclear. Klotho, a multifunctional protein with diverse biological activities, is primarily secreted into the circulatory system by peripheral organs and the choroid plexus in the brain. It binds to the FGFR1c receptor on hippocampal neurons and skeletal muscle fibers, thereby regulating neuronal and muscular function [5,6,7,8]. Accumulating evidence demonstrates an age-associated decline in Klotho levels [9]. Notably, elderly individuals with cognitive impairment exhibit significantly lower cerebrospinal fluid Klotho concentrations compared to their cognitively intact counterparts [9]. Furthermore, Klotho knockout in mice leads to reduced hippocampal long-term potentiation (LTP), contributing to cognitive deficits [10,11]. Klotho deficiency is also associated with physical dysfunction. Older adults with low Klotho levels report higher fatigue susceptibility and exhibit reduced lower-limb muscle mass [12]. Similarly, Klotho-deficient mice display growth retardation, decreased muscle mass, reduced body weight, and impaired mobility [13]. These findings collectively suggest that diminished circulating Klotho may serve as a key pathological contributor to CF, as it may represent a shared molecular link between hippocampal dysfunction and skeletal muscle impairment, the two major components of the syndrome.

To date, effective interventions for CF remain limited, highlighting an urgent need for further research. Our previous study found that aerobic exercise can enhance functional connectivity between the hippocampus and bilateral medial prefrontal cortex (mPFC) in older adults, improving cognitive function [14]. This finding provides an important rationale for including aerobic exercise in the present combined intervention strategy. Furthermore, sustained aerobic exercise improves lower-limb function in older adults [15]. Intriguingly, emerging evidence suggests that aerobic exercise may elevate serum Klotho levels in participants [16,17,18]. In recent years, multisensory stimulation has emerged as an effective intervention for cognitive deficits [19,20,21]. For instance, specific sensory modalities, such as auditory stimulation, have been shown to directly enhance behavioral performance and recovery. A recent study demonstrated that auditory beta-frequency binaural beats combined with preferred music significantly improved combat performance and recovery responses in athletes, providing a concrete example of how targeted sensory input can modulate brain function and behavioral outcomes [22].

Given that cognitive frailty is characterized by the concurrent decline of both motor and cognitive function, we hypothesize that Klotho deficiency may serve as a shared pathological nexus linking these dual impairments. Aerobic exercise may primarily counteract peripheral Klotho decline and improve muscle homeostasis, whereas multisensory stimulation may predominantly enhance central Klotho signaling and neural plasticity. Therefore, we posit that their combination may yield synergistic benefits surpassing either intervention alone, by concurrently augmenting systemic Klotho-dependent pathways. This rationale aligns with the design of multimodal practices such as yoga, which integrate physical exertion with sensory-cognitive engagement. Consequently, this study was designed not only to test the efficacy of combined training but, more specifically, to investigate the hypothesis that it ameliorates cognitive frailty through the synergistic modulation of systemic Klotho pathways in both the brain and skeletal muscle.

However, whether aerobic exercise combined with multisensory stimulation is superior to single-modality interventions for improving CF, and whether these effects are mediated through the Klotho pathway, remain to be further investigated.

## 2. Results

### 2.1. Aerobic Exercise Combined with Multisensory Stimulation Training Improves AT Motor and Cognitive Functions in CF Mice

**Figure 1:**
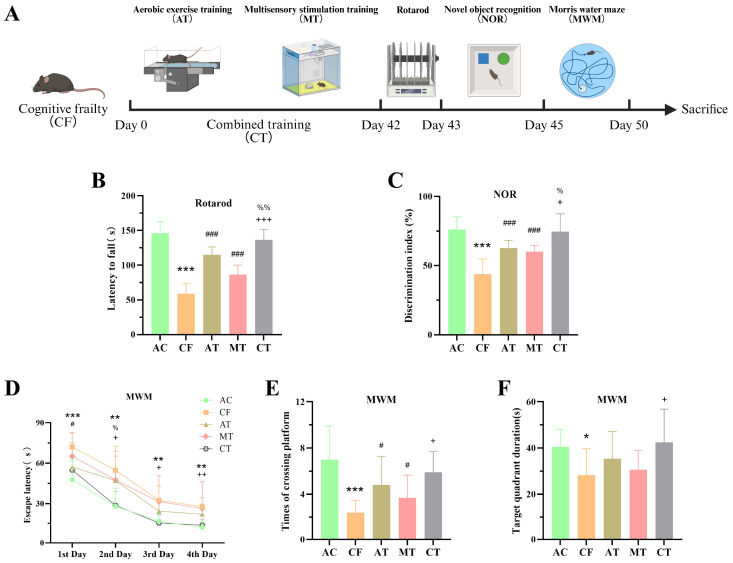
CT training rescues motor and cognitive deficits in CF mice. (**A**) A schematic diagram for the experimental paradigm to explore the effects of CT training in CF mice on the motion and cognition. (**B**) The latency to fall in the rotarod (n = 10 per group). (**C**) The discrimination index detected at 1 h after the learning stage in the novel object recognition test (n = 10 per group). (**D**–**F**) The latency to reach the escape platform, the number of crossings of the escape platforming and the duration percentage of the target quadrant in the MWM (n = 10 per group). Data are presented as means ± SD. Compared with AC group, * *p* < 0.05, ** *p* < 0.01, *** *p* < 0.001. Compared with CF group, ^#^ *p* < 0.05, ^###^ *p* < 0.001. Compared with MT group, ^+^ *p* < 0.05, ^++^ *p* < 0.01, ^+++^ *p* < 0.001. Compared with AT group, ^%^ *p* < 0.05, ^%%^ *p* < 0.01. AC, adult control; CF, cognitive frailty; AT, aerobic exercise training; MT, multisensory stimulation training; CT, combined training.

To investigate the effects of aerobic exercise and multisensory stimulation training on cognitively frail mice, we designed a training protocol as shown in Figure 1A. Results from the Rotarod test demonstrated that compared with adult control (AC) mice, cognitively frail (CF) mice exhibited a significantly shorter latency to fall off the rotarod. In contrast, mice in the aerobic exercise training (AT) group, multisensory stimulation training (MT) group, and combined training (CT) group all showed significantly prolonged rotarod retention times compared to the CF group (Figure 1B). These findings indicate that AT, MT and CT can improve muscle strength in CF mice.

In further behavioral tests, we found that AT, MT, and CT all increased the novel object recognition index in CF mice. In the Morris water maze, AT, MT, and CT reduced escape latency and increased the number of platform crossings, whereas only CT significantly increased the time spent in the target quadrant compared with CF mice (Figure 1C,D). Notably, the CT yielded superior effects compared to single-modality training. Collectively, these results suggest that aerobic exercise and multisensory stimulation training can improve both motor and cognitive functions in CF mice.

### 2.2. CT Improves Hippocampal Synaptic Plasticity and Skeletal Muscle Homeostasis in CF Mice

**Figure 2:**
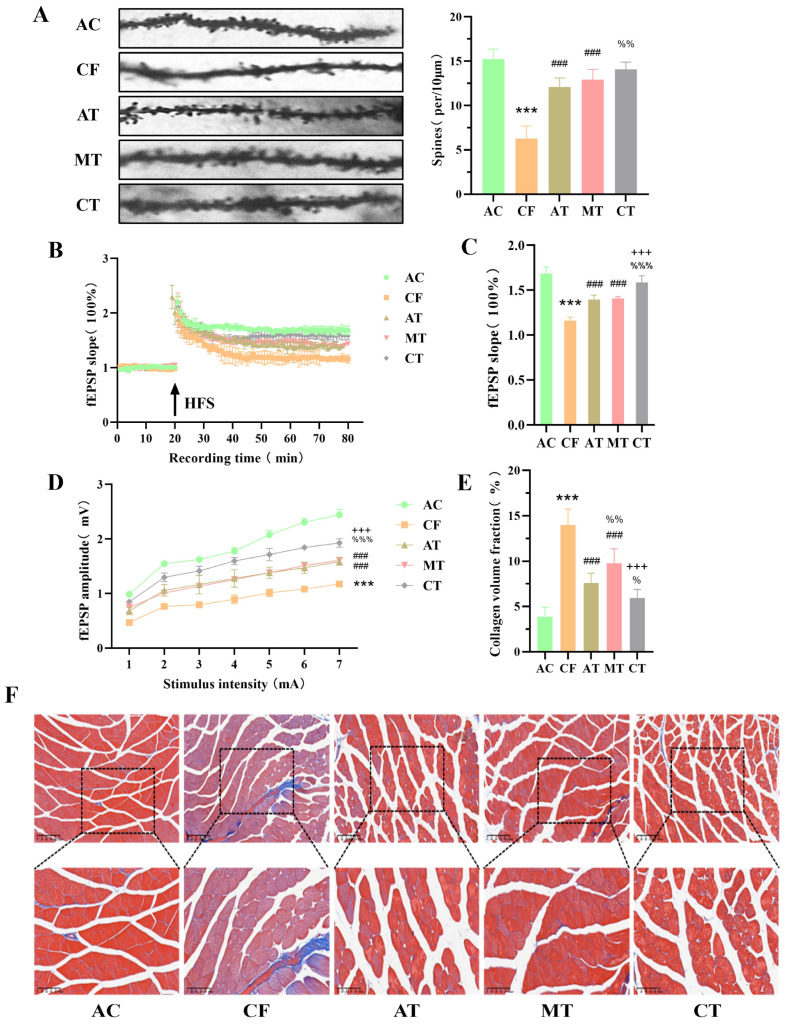
CT training improves synaptic plasticity in the hippocampus and skeletal muscle morphology of CF mice. (**A**) The representative images of Golgi staining in the hippocampus and the density of dendritic spine determined by Golgi staining in the hippocampus (n = 3 per group). (**B**) The time-plot of normalized fEPSP slope recorded from hippocampus. (**C**) The average normalized fEPSP slope during the last 10 min of recording following HFS in the hippocampus (n = 6 per group). (**D**) The I/O curve recordings in the hippocampus (n = 6 per group). (**E**) The collagen volume fraction in the skeletal muscle (n = 6 per group). (**F**) The representative images of Masson staining in the skeletal muscle. Compared with AC group, *** *p* < 0.001. Compared with CF group, ^###^ *p* < 0.001. Compared with MT group, ^+++^ *p* < 0.001. Compared with AT group, ^%^ *p* < 0.05, ^%%^ *p* < 0.01, ^%%%^ *p* < 0.001. AC, adult control; CF, cognitive frailty; AT, aerobic exercise training; MT, multisensory stimulation training; CT, combined training.

Golgi staining results revealed that AT, MT, and CT increased the density of hippocampal dendritic spines in CF mice (Figure 2A). Further electrophysiological experiments showed that AT, MT, and CT all enhanced hippocampal LTP and synaptic transmission efficiency (Figure 2B–D), with the combined training outperforming single-modality training. Taken together, these findings suggest that aerobic exercise combined with multisensory stimulation training effectively improves the structural and functional plasticity of hippocampal synapses in CF mice.

Subsequently, Masson staining showed that compared with CF mice, muscle fibers in the AT, MT and CT groups were more tightly arranged, with reduced collagen fiber deposition (Figure 2E,F) and decreased collagen volume fraction (Figure 2D), indicating that CT improves skeletal muscle homeostasis in CF mice.

### 2.3. CT Promotes the Expression of Klotho-Related Proteins in the Hippocampus of CF Mice

**Figure 3:**
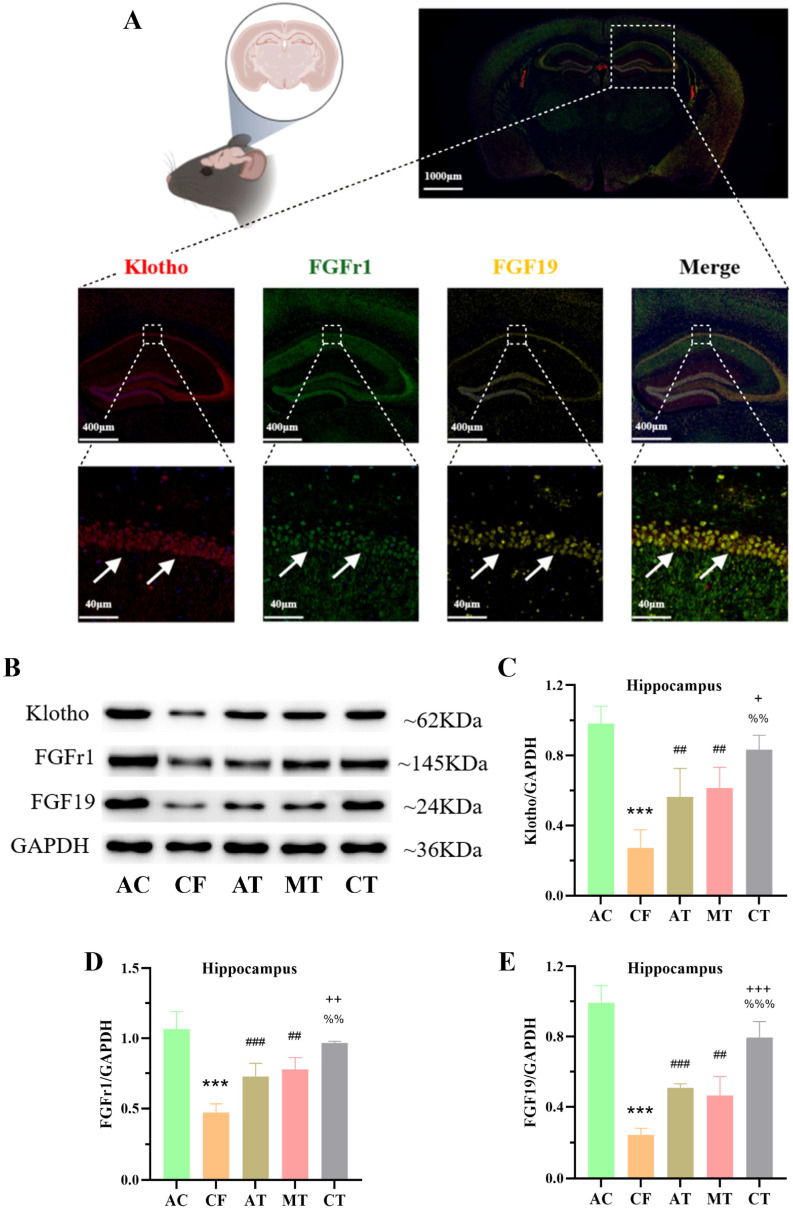
CT training regulates the expression of Klotho, FGFR1, FGF19 in the hippocampus of CF mice. (**A**) Immunofluorescent colocalization of Klotho, FGFR1 and FGF19 in the hippocampus. (**B**) The representative Western blots of Klotho, FGFR1 and FGF19 in the hippocampus. (**C**–**E**) The quantitative analysis of Klotho (**C**), FGFR1 (**D**) and FGF19 (**E**) in the hippocampus (n = 4 per group). Compared with AC group, *** *p* < 0.001. Compared with CF group, ^##^ *p* < 0.01, ^###^ *p* < 0.001. Compared with MT group, ^+^ *p* < 0.05, ^++^ *p* < 0.01, ^+++^ *p* < 0.001. Compared with AT group, ^%%^ *p* < 0.01, ^%%%^ *p* < 0.001. AC, adult control; CF, cognitive frailty; AT, aerobic exercise training; MT, multisensory stimulation training; CT, combined training.

Previous research has shown that in hippocampal neurons, circulating Klotho enhances synaptic plasticity by promoting FGF19-FGFR binding to activate downstream signaling and boost excitatory postsynaptic potentials, establishing its role as a critical regulatory protein [6,23]. In this study, immunofluorescence triple-labeling co-localization assays first revealed strong co-localization of Klotho, FGFR1, and FGF19 in the mouse hippocampus (Figure 3A). Further, Western blot (WB) analysis showed that AT, MT, and CT increased the expression of Klotho, FGF19, and FGFR1 in the hippocampus of CF mice (Figure 3B–E), with combined training further enhancing their expression compared to single-modality training. Additionally, co-localization of Klotho with the hippocampal neuronal marker Neun, microglial marker IBA-1, and astrocyte marker GFAP was observed in specific neuronal types (Figure S1). These results confirm that CT promotes the expression of Klotho-related proteins in the hippocampus of CF mice.

### 2.4. CT Promotes the Expression of Klotho-Related Proteins in the Gastrocnemius Muscle of CF Mice

**Figure 4:**
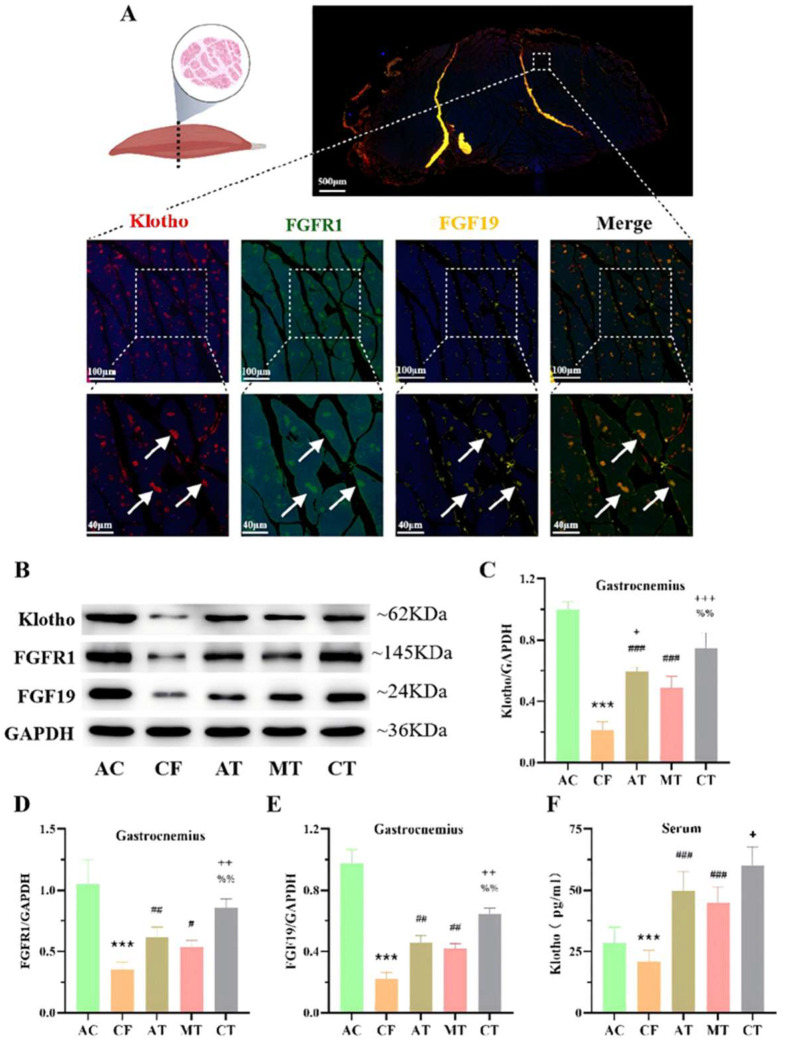
CT training regulates the expression of Klotho, FGFR1, FGF19 in the gastrocnemius of CF mice. (**A**) Immunofluorescent colocalization of Klotho, FGFR1 and FGF19 in the gastrocnemius. (**B**) The representative Western blots of Klotho, FGFR1 and FGF19 in the gastrocnemius. (**C**–**E**) The quantitative analysis of Klotho (**C**), FGFR1 (**D**) and FGF19 (**E**) in the gastrocnemius (n = 4 per group). (**F**) Elisa detection results of Klotho content in serum (n = 10 per group). Compared with AC group, *** *p* < 0.001. Compared with CF group, ^#^ *p* < 0.05, ^##^ *p* < 0.01, ^###^ *p* < 0.001. Compared with MT group, ^+^ *p* < 0.05, ^++^ *p* < 0.01, ^+++^ *p* < 0.001. Compared with AT group, ^%%^ *p* < 0.01. AC, adult control; CF, cognitive frailty; AT, aerobic exercise training; MT, multisensory stimulation training; CT, combined training.

Using immunofluorescence triple-labeling co-localization assays, we confirmed the co-expression of Klotho, FGF19, and FGFR1 in the gastrocnemius muscle (Figure 4A). Western blot analysis further showed that AT, MT, and CT increased the expression of Klotho, FGF19, and FGFR1 in the gastrocnemius muscle of CF mice (Figure 4B–E), with combined training further enhancing their expression compared to single-modality training. Interestingly, serum ELISA assays revealed that AT, MT and CT significantly increased serum Klotho levels in CF mice (Figure 4F). Studies have reported that circulating Klotho in skeletal muscle maintains skeletal muscle homeostasis and enhances motor function by promoting skeletal muscle fiber remodeling [24]. These results confirm that CT promotes the expression of Klotho-related proteins in the gastrocnemius muscle of CF mice.

Together, these findings demonstrate that CT activates a unified Klotho-mediated pathway in two key target tissues critical for cognitive frailty: the hippocampus and skeletal muscle. This coordinated upregulation suggests a systemic mechanism by which CT may concurrently address the central and peripheral aspects of the syndrome.

### 2.5. Inhibition of Systemic Klotho Expression Attenuates the Improvement of Motor and Cognitive Functions by CT in CF Mice

**Figure 5:**
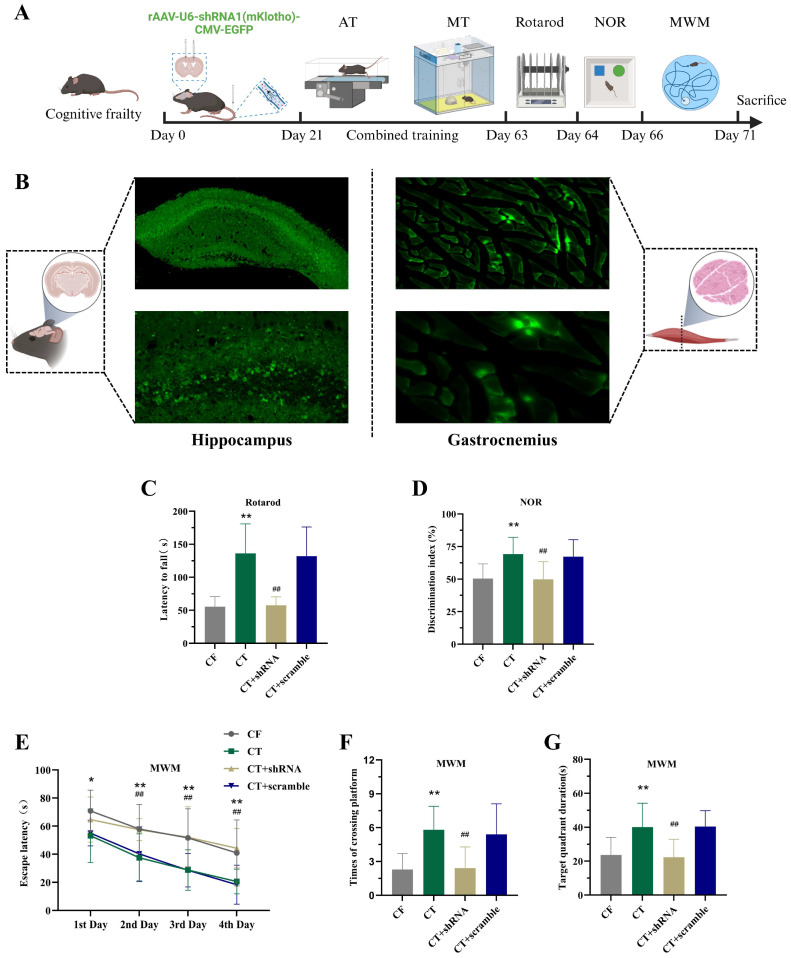
Inhibiting klotho in CF mice attenuates the improvement of cognitive and motor functions by CT training. (**A**) A schematic diagram for the experimental paradigm to explore the improvement of cognitive and motor function in CF mice through CT training mediated by klotho expression. (**B**) Schematic diagram of virus expression in the hippocampus and gastrocnemius muscle after Klotho inhibits virus injection. (**C**) The latency to fall in the rotarod (n = 10 per group). (**D**) The discrimination index detected at 1 h after the learning stage in the novel object recognition test (n = 10 per group). (**E**–**G**) The latency to reach the escape platform, the number of crossings of the escape platforming and the duration percentage of the target quadrant in the MWM (n = 10 per group). Data are presented as means ± SD. Compared with CF group, * *p* < 0.05, ** *p* < 0.01. Compared with CT + scramble group, ^##^ *p* < 0.01. CF, cognitive frailty model; AT, aerobic exercise training; MT, multisensory stimulation training; CT, combined training; NOR, novel object recognition; MWM, Morris water maze.

To investigate the critical role of Klotho in CT-mediated improvement of motor and cognitive functions in CF mice, we performed aerobic exercise combined with multisensory stimulation training while injecting Klotho shRNA lentivirus into the lateral ventricle and tail vein of mice (Figure 5A,B). In terms of motor function, compared with control virus-treated mice, Klotho-inhibited virus-treated mice showed decreased grip strength and rotarod retention time (Figure 5C). Further behavioral tests revealed that compared with control virus-treated mice, Klotho-inhibited virus-treated mice exhibited a reduced novel object recognition index, prolonged escape latency in the Morris water maze, decreased number of target platform crossings, and shorter duration in the target quadrant (Figure 5D,E). These results confirm that inhibiting systemic Klotho expression weakens the cognitive improvement effects of CT in CF mice.

### 2.6. Inhibition of Systemic Klotho Expression Attenuates CT-Mediated Improvement in Hippocampal Synaptic Structural Remodeling and Gastrocnemius Muscle Homeostasis in CF Mice

**Figure 6:**
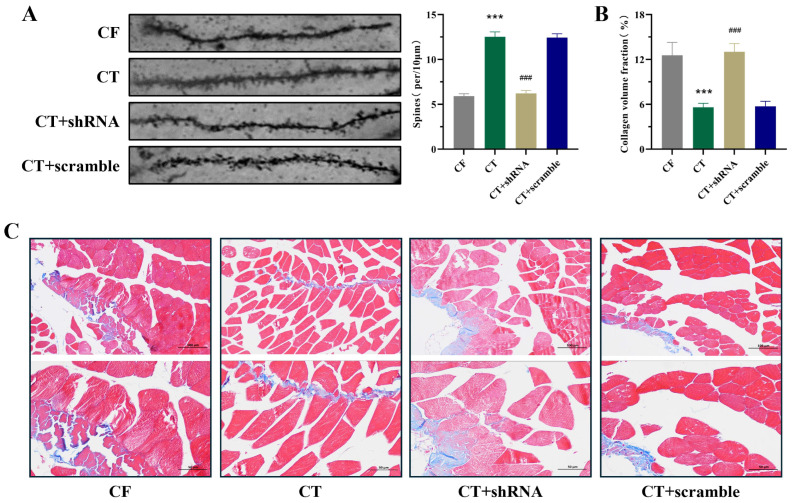
Inhibiting klotho in CF mice attenuates the improvement of hippocampal synaptic structure and skeletal muscle morphology. (**A**) The representative images of Golgi staining in the hippocampus and the density of dendritic spine determined by Golgi staining in the hippocampus (n = 3 per group). (**B**) The collagen volume fraction in the skeletal muscle (n = 6 per group). (**C**) The representative images of Masson staining in the skeletal muscle. Compared with CF group, *** *p* < 0.001. Compared with CT + scramble group, ^###^ *p* < 0.001.

To further clarify the effect of Klotho on hippocampal synaptic structure in CF mice, Golgi staining revealed that when systemic Klotho expression was inhibited, the density of hippocampal dendritic spines in CT-treated mice was significantly reduced (Figure 6A), confirming that inhibiting systemic Klotho expression weakens the beneficial effect of CT on hippocampal synaptic structure in CF mice. On the other hand, Masson staining showed that muscle fibers in the CT group had smaller gaps and less collagen fiber deposition, whereas Klotho-inhibited mice exhibited larger muscle fiber gaps and more collagen fiber deposition (Figure 6B,C). These results indicate that inhibiting systemic Klotho expression attenuates CT-induced improvement of gastrocnemius muscle homeostasis in CF mice.

### 2.7. Inhibition of Systemic Klotho Expression Impairs CT-Mediated Regulation of Hippocampal Synaptic Plasticity and Skeletal Muscle Homeostasis-Related Proteins in CF Mice

**Figure 7:**
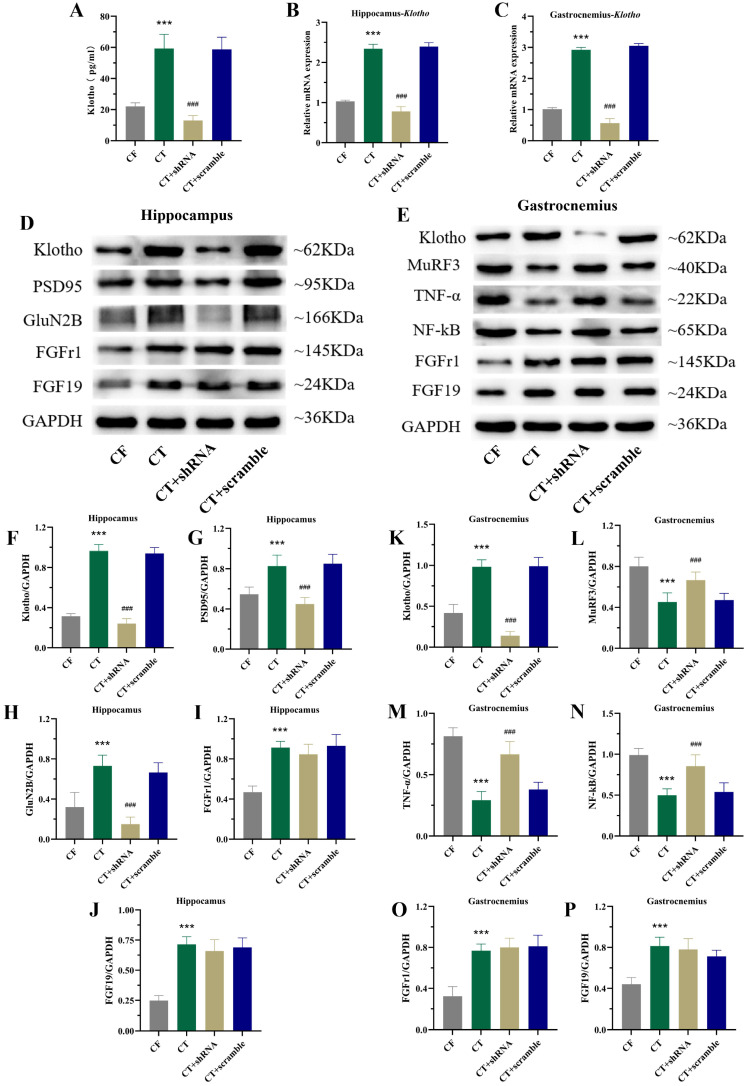
Expression results of klotho-related proteins in hippocampus and gastrocnemius after injecting klotho-inhibiting virus in CF mice. (**A**) The detection of Klotho content in serum, hippocampus and the gastrocnemius (n = 4 per group). (**B**) The representative Western blots of Klotho, PSD95, GluN2B, FGFR1 and FGF19 in the hippocampus. (**C**) The representative Western blots of Klotho, MuRF3, TNF-α, NF-κB, FGFR1 and FGF19 in the gastrocnemius. (**D**) The quantitative analysis of Klotho, PSD95, GluN2B, FGFR1 and FGF19 in the hippocampus (n = 4 per group). (**E**) The quantitative analysis of Klotho, MuRF3, TNF-α, NF-κB, FGFR1 and FGF19 in the gastrocnemius (n = 4 per group). (**F**–**P**) Protein result statistics chart. Compared with CF group, *** *p* < 0.001. Compared with CT + scramble group, ^###^ *p* < 0.001. CF, cognitive frailty model; CT, combined training.

The study further examined the expression of functional proteins regulating hippocampal synaptic plasticity and skeletal muscle homeostasis in CT-treated mice after inhibiting systemic Klotho. Following injection of Klotho shRNA lentivirus during CT training, serum Klotho levels were significantly reduced (Figure 7A), and Klotho mRNA expression levels in both the hippocampus and gastrocnemius muscle were also markedly decreased (Figure 7A). Western blot results showed that compared with control virus-treated mice, Klotho-inhibited virus-treated mice exhibited decreased expression of synaptic plasticity-related proteins PSD95 and GluN2B in the hippocampus (Figure 7B,D). Additionally, Klotho-inhibited mice showed increased expression of MuRF3, TNF-α, and NF-κB in the gastrocnemius muscle (Figure 7C,E). These results indicate that inhibiting systemic Klotho expression impairs CT-induced regulation of proteins associated with hippocampal synaptic plasticity and skeletal muscle homeostasis in CF mice.

## 3. Discussion

### 3.1. Aerobic Exercise Combined with Multisensory Stimulation Contributes to the Recovery of Functional Deficits in Cognitive Frailty

In this study, we designed a training protocol combining aerobic exercise and multisensory stimulation to intervene in cognitively frail mice. We found that both AT and MT improved cognitive function in cognitively frail mice, consistent with clinical findings that meditation or exercise training improve cognitive outcomes in patients with cognitive impairment [25,26,27,28]. Notably, our study revealed that CT produced more pronounced improvements in cognitive function compared to AT or MT alone, aligning with a study in JAMA Network Open showing that exercise combined with cognitive training outperforms single-modality interventions for cognitive improvement in patients with mild cognitive impairment (MCI) [29]. Additionally, in terms of motor function, AT and MT both increased the retention time of cognitively frail mice in the rotarod test, with CT demonstrating superior effects. Collectively, these findings suggest that aerobic exercise combined with multisensory stimulation may be a promising intervention for CF.

### 3.2. CT Regulates Functional Recovery in Cognitive Frailty by Modulating Klotho Expression

The Klotho protein, encoded by the Klotho gene, serves as an anti-aging and longevity factor that functions as both a transmembrane protein and circulating factor. Its expression declines with age, contributing to various age-related pathologies, and interventions such as dietary regimens and physical training have been shown to counteract this decline and associated dysfunction [30,31]. It plays a pivotal role in cellular metabolism and systemic homeostasis, while also participating in the development and regulation of the central nervous and skeletal muscle systems [32,33,34]. A landmark study published in Nature demonstrated that Klotho-knockout mice exhibit aging-associated phenotypes by 4–5 weeks postnatally, characterized by growth retardation, muscle atrophy, and neuronal damage [35]. Further investigations revealed that 7-week-old Klotho-deficient mice display impaired hippocampal neurogenesis, marked by reduced neural stem cells and immature neurons, ultimately leading to spatial learning and memory deficits [36]. Numerous clinical and preclinical studies have established that aerobic exercise training elevates systemic Klotho levels, thereby improving neurological function [16,17,18,37]. Notably, a randomized controlled trial employing transcriptomic analysis found that 8 weeks of yoga training (combining aerobic exercise and multisensory stimulation) significantly increased serum Klotho mRNA levels in sedentary individuals [38].

Here, we demonstrated that both AT and multisensory MT increased Klotho protein levels in serum and skeletal muscle, with CT yielding further enhancement compared to MT alone. Although CT showed a trend toward higher Klotho levels than AT, this difference did not reach statistical significance. This observation may be attributed to the fact that serum and skeletal muscle Klotho primarily originates from skeletal muscle and visceral organs. Skeletal muscle is a direct target organ of AT, whereas MT may induce central nervous system-driven skeletal muscle responses by regulating brain rhythms, promoting Klotho protein release. CT, by combining central and peripheral regulation, produces a more pronounced increase in Klotho protein. In the hippocampus, both AT and MT increased Klotho expression, with CT demonstrating superior efficacy. Furthermore, immunofluorescence co-localization experiments confirmed strong spatial overlap between Klotho, FGF19, and FGFR1 in hippocampal and skeletal muscle tissues, suggesting signal transduction occurs through formation of the FGF-FGFR-Klotho ternary complex.

To determine whether CT improves functional deficits in cognitively frail mice via systemic Klotho, we administered Klotho shRNA lentivirus via intracerebroventricular and tail vein injections to suppress systemic Klotho expression in CF mice. Compared with the CT group, the Klotho-inhibited group exhibited prolonged escape latency, reduced platform crossings, shorter target quadrant residence time in the Morris water maze, decreased novel object recognition index, and reduced rotarod retention time. These results demonstrate that CT improves cognitive and motor functions by regulating Klotho expression.

### 3.3. CT-Mediated Klotho Expression Regulates Skeletal Muscle Homeostasis and Hippocampal Synaptic Plasticity

Previous studies have established that the FGF19-FGFR1-Klotho ternary complex plays a pivotal role in regulating skeletal muscle inflammation and fiber remodeling, thereby maintaining muscle homeostasis [39]. In this study, AT, MT, and CT all increased the protein levels of FGF19 and its specific receptor FGFR1 in the hippocampus and gastrocnemius muscle, providing a material basis for the formation of the FGF19-FGFR1-Klotho ternary complex and promoting Klotho signaling. Notably, existing literature indicates that Klotho can interact with IκBα, a critical regulator of the NF-κB pathway, to suppress the production of pro-inflammatory cytokines [40].

The results showed that compared with the CT group, the Klotho-inhibited group exhibited decreased Klotho protein expression in the gastrocnemius muscle, accompanied by increased expression of MuRF3, NF-κB, and TNF-α. Masson staining further revealed that Klotho-inhibited mice had loosely arranged muscle fibers, increased inter-fiber spacing, more collagen fiber deposition, and poor gastrocnemius morphology compared with CT-treated mice. These findings confirm that CT enhances skeletal muscle homeostasis and improves muscle fiber morphology by upregulating systemic Klotho.

Studies have reported that Klotho-overexpressing mice exhibit better cognitive function than age-matched wild-type mice, with increased brain expression of GluN2B and PSD95, which enhance synaptic AMPAR channel currents [33]. A recent study showed that exogenous Klotho supplementation in aging rhesus macaques improved cognitive function and maintained this effect for an extended period [41]. Another study found that knocking down Klotho in the nucleus accumbens led to cognitive behavioral abnormalities in mice, accompanied by decreased PSD95 and GluN2B expression in the nucleus accumbens [10]. These results suggest that Klotho may improve aging-related cognitive decline by mediating GluN2B expression to increase the thickness of postsynaptic density.

### 3.4. An Integrative Model: Klotho as a Systemic Coordinator

Our findings support a model wherein CT elevates systemic Klotho, which then acts as a key humoral mediator coordinating multi-system improvements. A critical, unresolved question is how CT upregulates Klotho expression. We speculate that this may involve the amelioration of upstream age-related drivers, such as chronic low-grade inflammation and metabolic dysregulation, both known suppressors of Klotho expression and both positively influenced by exercise and environmental enrichment. Future studies should directly test whether CT reduces inflammatory cytokines (e.g., IL-6, TNF-α) or improves insulin sensitivity, and how these changes correlate with Klotho induction.

Collectively, our findings support an integrative model wherein CT elevates systemic Klotho, which then acts as a pivotal humoral coordinator of multi-system improvements. The central question of how CT upregulates Klotho warrants further investigation but may involve the amelioration of upstream age-related drivers such as chronic inflammation and metabolic dysregulation, both known suppressors of Klotho expression. Furthermore, we propose that increased circulating Klotho serves as a critical messenger that synchronously addresses the dual facets of cognitive frailty. In the hippocampus, Klotho enhances synaptic plasticity, potentially by stabilizing the NMDAR subunit GluN2B and promoting the scaffold protein PSD95, thereby strengthening synaptic transmission and supporting cognitive function. Concurrently, in skeletal muscle, Klotho exerts anti-inflammatory and antioxidant effects, likely by interfering with the NF-κB pathway, as supported by its established role in mitigating oxidative stress and inflammation in age-related conditions [42], leading to reduced expression of atrophy-related factors like TNF-α and MuRF3, thereby preserving muscle homeostasis and function. This dual-tissue, Klotho-centered mechanism provides a plausible explanation for the synergistic recovery of motor and cognitive functions observed with CT, positioning Klotho not merely as a biomarker but as a central orchestrator of systemic resilience against age-related decline.

### 3.5. Limitations of the Study

While this study provides mechanistic insights into the synergistic effects of combined training, several limitations should be acknowledged, which also point to valuable directions for future research.

First, regarding the experimental model, the use of a young adult control group, while standard for establishing a baseline of age-related decline [43], precludes a precise dissection of deficits attributable specifically to the cognitive frailty phenotype versus natural aging. However, our primary conclusions about the superior efficacy of the combined intervention are robust, as they are derived from direct comparisons within age-matched cohorts. Future studies incorporating an aged, untreated control group would be beneficial.

Second, the specificity and scope of our mechanistic validation could be enhanced. The shRNA-mediated knockdown was systemic rather than tissue- or cell-type-specific, limiting our ability to pinpoint the exact cellular sources of Klotho critical for functional recovery. Furthermore, while we confirmed knockdown in serum, hippocampus, and muscle, direct validation in major Klotho-producing organs like the kidneys was not performed. Employing conditional knockout models and multi-tissue analysis in future work will provide more precise mechanistic insights.

Third, certain intervention parameters and the study cohort present opportunities for refinement. Similarly, the individual contributions and optimal parameters of the multisensory stimulation components warrant systematic deconstruction in future studies.

Another limitation of the present study is that only male mice were included. Although this design helped reduce biological variability and improve mechanistic interpretability in this initial proof-of-concept study, it may limit the generalizability of our findings. Future studies should include female mice to determine whether the Klotho-mediated effects of combined training differ by sex.

## 4. Materials and Methods

### 4.1. Experimental Animals and Grouping

Male C57BL/6 mice (Slaccas, Shanghai, China; License No.: SCXK[H]2017-0005) were housed under specific pathogen-free conditions at the Laboratory Animal Center of Fujian University of Traditional Chinese Medicine (License No.: SYXK[M]2020-0002). Animals were maintained at 22 ± 1 °C with 50 ± 5% humidity under a 12:12 h light-dark cycle, with ad libitum access to standard chow and water. Male mice were used in this study to reduce biological variability in this initial mechanistic investigation and to facilitate interpretation of the effects of combined training on the systemic Klotho-centered pathway under a relatively controlled experimental background. All animal procedures were approved by the Institutional Animal Care and Use Committee of Fujian University of Traditional Chinese Medicine (FJTCM IACUC 2022045) and were conducted in accordance with the relevant institutional guidelines and the International Guidelines for Animal Care and Use.

In this experiment, 6-month-old C57BL/6 mice served as the adult control group, and 16-month-old mice were the senescent group. The use of 6-month-old mice as the healthy adult control is a well-established design in aging research using C57BL/6 mice, providing a clear baseline of young adult physiology against which age-related decline can be assessed [44,45]. This approach has been employed in our recent published work [43]. The 16-month-old mice were employed as a model of age-related cognitive frailty (CF). This model is supported by established literature where C57BL/6 mice at this age exhibit concurrent motor and cognitive deficits, and it has been successfully used in our recent study investigating similar functional decline [43]. In the present cohort, the CF phenotype was operationally confirmed by the significant behavioral impairments observed in the CF group compared to the young adult control group. Specifically, CF mice exhibited impaired motor coordination as indicated by a significantly shorter latency to fall in the rotarod test, and deficits in cognitive function, as evidenced by a decreased discrimination index in the novel object recognition (NOR) task and prolonged escape latency, fewer platform crossings, and reduced time spent in the target quadrant in the Morris water maze (MWM), suggesting impairments in spatial learning and memory (Figure 1B–D). These mice were randomly divided into four groups: the age-related cognitive frailty (CF) group, the aerobic exercise training group (AT), the multisensory stimulation training (MT) group, and the combined training (CT) group, with 12 mice in each group. The CT group received both aerobic exercise and multisensory stimulation training.

### 4.2. Intervention Program

The experimental interventions, detailed below, were maintained for 6 consecutive weeks. Post-intervention, all animals were returned to their standard housing conditions.

#### 4.2.1. Aerobic Exercise Training

A forced aerobic exercise regimen was implemented using a motorized treadmill with only minor modifications, based on the protocol described by Lin et al. [43]. The detailed protocol included a 5-day acclimation phase and the 6-week training regimen comprising warm-up, main exercise, and cool-down periods. In brief, mice underwent moderate-intensity running at 12 m/min for 50 min per day, 5 days per week, for 6 consecutive weeks. Age-matched control (CF) mice were placed on a stationary treadmill for an equivalent duration under identical environmental conditions to control for handling and placement stress.

#### 4.2.2. Multisensory Stimulation Training

Multisensory stimulation was administered within an enriched environment modified from an established protocol. Individual mice were placed in a rectangular chamber (80 × 50 × 35 cm) lined with corncob bedding and equipped with a simulated cave containing sawdust. The stimulation explicitly included: (1) tactile enrichment via varied bedding materials (corncob and sawdust within a simulated cave); (2) visual modulation through sustained red light illumination (intensity ≤ 10 lux) creating a semi-dark environment; and (3) spatial/exploratory stimulation provided by the size of the arena itself and the presence of the cave structure. The chamber environment was rigorously maintained at a temperature of 23 ± 2 °C, relative humidity of 55 ± 3% (monitored and controlled using a calibrated digital thermohygrometer, Fluke Calibration, Everett, WA, USA), and under red light illumination (intensity ≤ 10 lux). Animal locomotion was quantified throughout the 120 min daily session using an overhead video tracking system.

#### 4.2.3. Combined Training

Combined training: Mice assigned to the combined group underwent the aerobic exercise training immediately prior to the multisensory stimulation training each day. The specific parameters for each component were as described in Section 4.2.1 and Section 4.2.2, respectively. This sequential protocol, with a total daily duration of 3 h, was scheduled between 8:00 a.m. and 4:00 p.m.

### 4.3. Behavioral Testing

Novel object recognition: The novel object recognition task was performed to assess recognition memory. The procedure consisted of a training phase and a testing phase. In the training phase, two identical objects (A1 and A2), which were green conical blocks, were placed at fixed positions within the apparatus. Objects A1 and A2 were made of plastic and had the same size and shape. The objects were cleaned with 75% ethanol before each session to eliminate potential odor cues. Each mouse was allowed to explore freely for 10 min, after which it was returned to its home cage. Following a 1 h interval, the mouse was reintroduced to the apparatus for a 5 min session, during which object A2 was replaced with a novel object B, a red cylindrical block of similar size and material to the conical objects, but with a distinct shape and color to ensure novelty. All sessions were video-recorded for subsequent analysis. The exploration time directed towards the familiar object (A1, recorded as Ta) and the novel object (B, recorded as Tb) was measured. A recognition index was calculated as follows: Recognition Index (%) = [Tb/(Ta + Tb)] × 100%.

Morris water maze: The Morris Water Maze was used to evaluate spatial learning and memory. The test comprised a 4-day acquisition trial followed by a probe trial. Over four consecutive days, each mouse underwent four training trials per day from four different entry points. For each trial, the mouse was given 90 s to locate a submerged platform. The time taken to find the platform was recorded as the escape latency. If a mouse failed to find the platform within 90 s, it was gently guided to it, and an escape latency of 90 s was recorded; the mouse was then allowed to remain on the platform for 15 s. Twenty-four hours after the final acquisition trial, a probe trial was conducted to assess spatial memory retention. The platform was removed, and each mouse was released from the quadrant opposite the original platform location and allowed to swim freely for 90 s. The number of crossings over the former platform location and the time spent in the target quadrant were recorded. The timeline for all behavioral tests, including the multi-day MWM protocol, is explicitly summarized in Figure 1A. Specifically, the acquisition trials spanned four consecutive days (Days 45–48), followed by the probe trial on Day 49.

Rotarod test: Motor coordination and balance were evaluated using the rotarod test. The apparatus (model 47600, Ugo Basile, Comerio, Italy) consisted of five rotating cylinders (3 cm diameter, knurled surface) arranged in five lanes (5.7 cm wide each), enabling five mice to be tested simultaneously. The rotating rod was positioned 16 cm above the base plate, with a soft foam pad placed at the bottom to cushion falls and minimize potential stress or injury. During the test, the rod rotated at a constant speed of 20 rotations per minute (rpm). The latency to fall was recorded. Each mouse underwent three trials with a 10 min inter-trial rest interval. The average latency of the three trials was calculated for analysis. The rotarod test was performed as the first behavioral assessment to avoid carry-over effects on subsequent cognitive tests.

### 4.4. Immunofluorescence Co-Labeling

Paraformaldehyde-fixed mouse brain tissues were embedded in OCT compound (Thermo Fisher Scientific, Waltham, MA, USA), snap-frozen in liquid nitrogen, and sectioned into 5 μm thick coronal slices. For staining, the sections were first thawed for 30 min, followed by washes with PBST and PBS. Permeabilization was performed using 0.3% Triton X-100 at room temperature for 20 min. After PBS rinses, the sections were blocked with 5% BSA for 1 h at room temperature. Subsequently, a proportional mixture of primary antibodies against Klotho, FGFR1, and FGF19 was applied, and the sections were incubated at 4 °C for over 16 h. The following day, after PBS washes, the sections were incubated with a combination of two differently fluorophore-conjugated secondary antibodies from distinct sources for 1 h at room temperature protected from light. Finally, after further PBS washes, the sections were mounted with an anti-fade medium containing DAPI for nuclear staining.

### 4.5. Masson Staining

The staining procedure for Masson’s trichrome was performed as follows. After sectioning, nuclei were stained with Weigert’s iron hematoxylin for 5–10 min. The sections were then differentiated in an ethanol solution for 20 s and rinsed under running water for 25 min. Subsequently, they were stained with Masson’s Ponceau–Fuchsine solution for 5–10 min, rinsed in a weak acid solution for 1 min, and treated with 1% phosphomolybdic acid aqueous solution for 3–5 min for differentiation. After another 1 min rinse in weak acid solution, the sections were counterstained with aniline blue solution for 1–2 min, followed by a final 1 min weak acid rinse. Finally, the sections were dehydrated, cleared in xylene, and mounted with neutral resin.

### 4.6. Golgi Staining

Dendritic spines were visualized using the FD Rapid Golgi Stain Kit. Briefly, 24 h before tissue collection, Solutions A and B were mixed and allowed to settle. Immediately after extraction, the brain was cut into 1 cm thick coronal blocks and immersed in the mixed A/B solution for 14 days in the dark at room temperature. The tissue was then transferred to Solution C and stored under the same conditions for an additional 5 days. Subsequently, 100 μm thick sections were prepared using a cryostat, mounted on slides pre-treated with Solution C, and stained by immersion in a working solution containing Solutions D, E, and distilled water. After two rinses in distilled water, the sections were dehydrated in a graded ethanol series (50%, 75%, 95%), cleared in xylene, and coverslipped with neutral resin. The slides were stored in a dark, dry environment. Stained tissues were observed and imaged using an intelligent slide scanning system (Aperio VERSA, Leica, Germany).

### 4.7. Electrophysiological Testing

Hippocampal and prefrontal cortical (PFC) slices (400 μm thick) were prepared in ice-cold, high-sucrose artificial cerebrospinal fluid (ACSF). Following sectioning, slices were recovered and continuously perfused with oxygenated normal ACSF at room temperature for at least 2 h before recording.

Field excitatory postsynaptic potentials (fEPSPs) were recorded using a microelectrode system. For hippocampal recordings, a bipolar stimulating electrode was positioned in the Schaffer collateral pathway within the CA3 region, and a glass recording electrode was placed in the stratum radiatum of the CA1 region. For PFC recordings, the stimulating electrode was positioned in layer V and the recording electrode in layers II/III.

Upon contact with the ACSF, the recording electrode was switched to current-clamp mode (I = 0), and the liquid junction potential was adjusted to approximately 0 mV. Electrode resistance was confirmed to be within 3–6 MΩ. The input–output (I/O) relationship was first established by applying stimuli of increasing intensity (10–70 μA in 10 μA steps). For each intensity, three responses were averaged.

The stimulus intensity evoking 30% of the maximum fEPSP slope was selected for baseline recording. After a 20 min stable baseline was established (with a single stimulus delivered every 20 s), long-term potentiation (LTP) was induced by applying two trains of high-frequency stimulation (100 Hz for 1 s) at the same intensity, with an inter-train interval of 30 s. Synaptic responses were then recorded for an additional 60 min to monitor LTP expression.

### 4.8. Viral Injection

To systemically inhibit Klotho expression in both the central nervous system and peripheral tissues, we employed a dual-route administration strategy. Klotho shRNA-expressing lentivirus was delivered via intracerebroventricular injection to target brain-derived Klotho, and concurrently via tail vein injection to target Klotho in peripheral organs, such as the kidneys and skeletal muscle, which are major contributors to circulating Klotho levels.

Twenty-one days prior to intervention, Klotho shRNA-expressing viral vectors (rAAV-U6-shRNA(mKlotho)-CMV-EGFP) or control scrambled shRNA vectors (rAAV-U6-shRNA(Scramble)-CMV-EGFP) were stereotactically delivered into the lateral ventricles and systemically administered via tail vein injection in the experimental and control groups, respectively.

Stereotactic intracerebroventricular injection: Mice were anesthetized with 3% isoflurane and secured in a stereotaxic frame under sustained anesthesia (1% isoflurane). After scalp shaving and midline incision, bregma was identified as the coordinate origin (0, 0, 0). Bilateral ventricular coordinates (±1.2 mm lateral, −0.35 mm posterior, −2.5 mm ventral relative to bregma) were determined according to the Allen Brain Atlas. A microdrill created cranial openings above target sites, through which 10 μL viral suspension was infused into each lateral ventricle via microsyringe at 1 μL/min. The needle was withdrawn slowly post-injection, followed by wound suturing and recovery in a thermoregulated chamber before returning to home cages.

Tail vein systemic delivery: Isoflurane-anesthetized (3% induction, 1% maintenance) mice were positioned in a tail vein injector. After alcohol swab disinfection, 20 μL viral solution was administered intravenously via insulin syringe. Hemostasis was achieved by brief cotton compression post-needle withdrawal prior to recovery and cage return.

### 4.9. RT-qPCR

Total RNA was extracted from tissues using the TRIzol method, and then reverse transcription and fluorescent quantitation reactions were performed according to the corresponding kits for miRNA and mRNA. Quantitative PCR was conducted using a CFX Opus 96 Real-Time PCR System (Bio-Rad, Hercules, CA, USA). Relative quantitative analysis was used, subtracting the Ct value of the reference gene from the Ct value of the target gene in the same sample to obtain ΔCt, which represented the expression intensity of the target gene relative to the reference gene. The 2^−ΔΔCt^ method was then used to express the expression difference of the target gene as the multiple of the treated sample relative to the untreated sample.

### 4.10. Western Blot

Hippocampal and gastrocnemius tissues were lysed in RIPA buffer (Boster (Pleasanton, CA, USA), AR0102-100) containing 1 mM PMSF (Boster, AR1192), homogenized by sonication, and quantified using a BCA protein assay. A total of 25 μg protein per lane was separated by SDS-PAGE using a Mini-PROTEAN electrophoresis system (Bio-Rad, Hercules, CA, USA) and transferred onto PVDF membranes using a Mini Trans-Blot transfer system (Bio-Rad, Hercules, CA, USA). Membranes were blocked with 5% non-fat milk and incubated overnight at 4 °C with the following primary antibodies: anti-Klotho (Abcam (Cambridge, UK), ab181373, 1:1000), anti-FGF19 (Abcam, ab225942, 1:3000), anti-FGFR1 (CST (Danvers, MA, USA), 9740, 1:1000), anti-PSD95 (Proteintech (Rosemont, IL, USA), 20665-1-AP, 1:10,000), anti-GluN2B (Proteintech, 21920-1-AP, 1:2000), anti-MuRF3 (Abcam, ab172479, 1:10,000), anti-NF-κB (Proteintech, 10745-1-AP, 1:3000), anti-TNF-α (Proteintech, 60291-1-Ig, 1:2000), and anti-GAPDH (Proteintech, 60004-1-Ig, 1:10,000). After washing with TBST, membranes were incubated with HRP-conjugated goat anti-mouse or goat anti-rabbit IgG secondary antibodies (1:5000) at room temperature for 1 h. Protein bands were detected using a ChemiDoc XRS+ Imaging System (Bio-Rad, Hercules, CA, USA), and band intensities were normalized to GAPDH.

### 4.11. Enzyme-Linked Immunosorbent Assay

Blood was collected from the heart after anesthesia, allowed to clot at room temperature for 1 h, and then centrifuged at 3000 rpm for 15 min to obtain serum. Serum Klotho levels were measured using a commercial mouse Klotho ELISA kit (Elabscience (Houston, TX, USA), E-EL-M3051) according to the manufacturer’s instructions. Briefly, 100 μL of diluted serum samples and standards were added to pre-coated plates and incubated at 37 °C for 90 min. Subsequently, 100 μL of biotinylated detection antibody was added and incubated at 37 °C for 60 min, followed by incubation with 100 μL of HRP-conjugated streptavidin at 37 °C for 30 min. Finally, 90 μL of TMB substrate was added and incubated in the dark for 15 min at room temperature. The reaction was stopped with 50 μL of stop solution, and absorbance was measured at 450 nm using a Tecan Spark microplate reader (Tecan, Männedorf, Switzerland). Klotho concentrations were determined from a standard curve, generated by a four-parameter logistic curve fit.

### 4.12. Statistical Analysis

In this study, SPSS 25.0 software was used for statistical analysis of all experimental data. Normality of data distribution was assessed using the Shapiro–Wilk test, and homogeneity of variances was evaluated using Levene’s test. Data are presented as mean ± SD. Repeated-measures ANOVA was applied to analyze the I/O curve and escape latency in the Morris water maze, while one-way ANOVA was used for the other experimental data. When variances were homogeneous, pairwise comparisons were performed using the LSD test. If variances were not homogeneous, Dunnett’s T3 test was used for pairwise comparisons. A value of *p* < 0.05 was considered statistically significant.

## Data Availability

The data that support the findings of this study are available from the corresponding authors upon reasonable request.

## Supplementary Materials

The following supporting information can be downloaded at: https://www.mdpi.com/article/10.3390/ijms27093991/s1.

## Abbreviations

The following abbreviations are used in this manuscript:

CF	Cognitive frailty
LTP	Long-term potentiation
mPFC	Medial prefrontal cortex
AC	Adult control
AT	Aerobic exercise training
MT	Multisensory stimulation training
CT	Combined training
MCI	Mild cognitive impairment
ACSF	Artificial cerebrospinal fluid
fEPSPs	Field excitatory postsynaptic potentials
