# Supplementary material for: Aerobic Exercise Combined with Multisensory Stimulation Training Improves Cognitive Frailty by Modulating Circulating Klotho
Source: Int J Mol Sci. 2026 Apr 29;27(9):3991. doi: 10.3390/ijms27093991 (PMC13163708; doi:10.3390/ijms27093991)
Supplement: Supplementary file 1 [file ijms-27-03991-s001.zip › Figure S1.pdf]

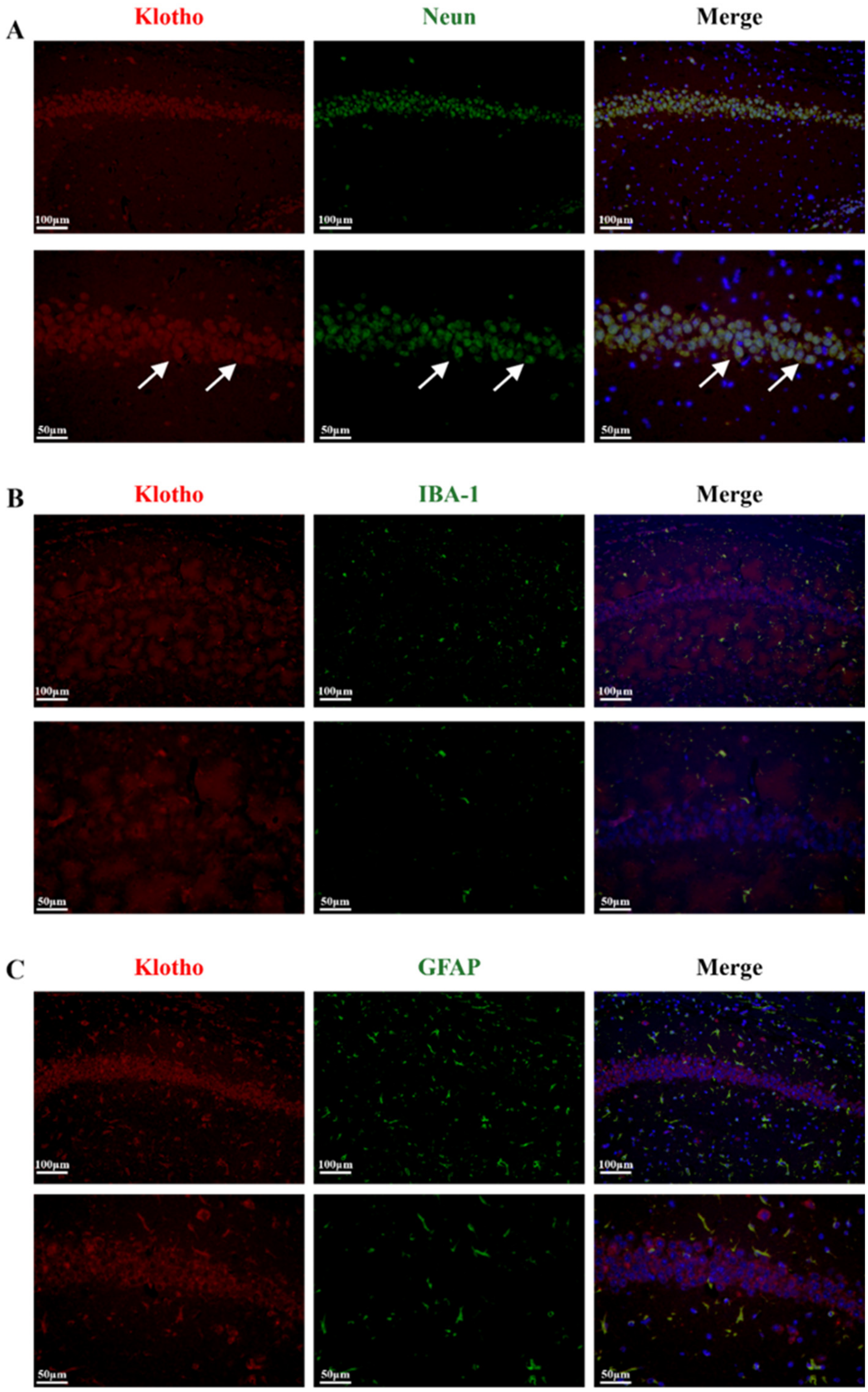

Figure S1. Immunofluorescence colocalization results of Klotho with different neural markers in hippocampus . (A) Immunofluorescence colocalization results of Klotho and neuronal marker Neun. **(B)** Immunofluorescence colocalization results of Klotho and microglia's marker IBA-1. **(C)** Immunofluorescence colocalization results of Klotho and astrocyte's marker GFAP.
