# Supplementary material for: Aerobic Exercise Combined with Multisensory Stimulation Training Improves Cognitive Frailty by Modulating Circulating Klotho
Source: Int J Mol Sci. 2026 Apr 29;27(9):3991. doi: 10.3390/ijms27093991 (PMC13163708; doi:10.3390/ijms27093991)
Supplement: Supplementary file 1 [file ijms-27-03991-s001.zip › Raw data of Western Blot.pdf]

Original gel images corresponding to Figure 3B

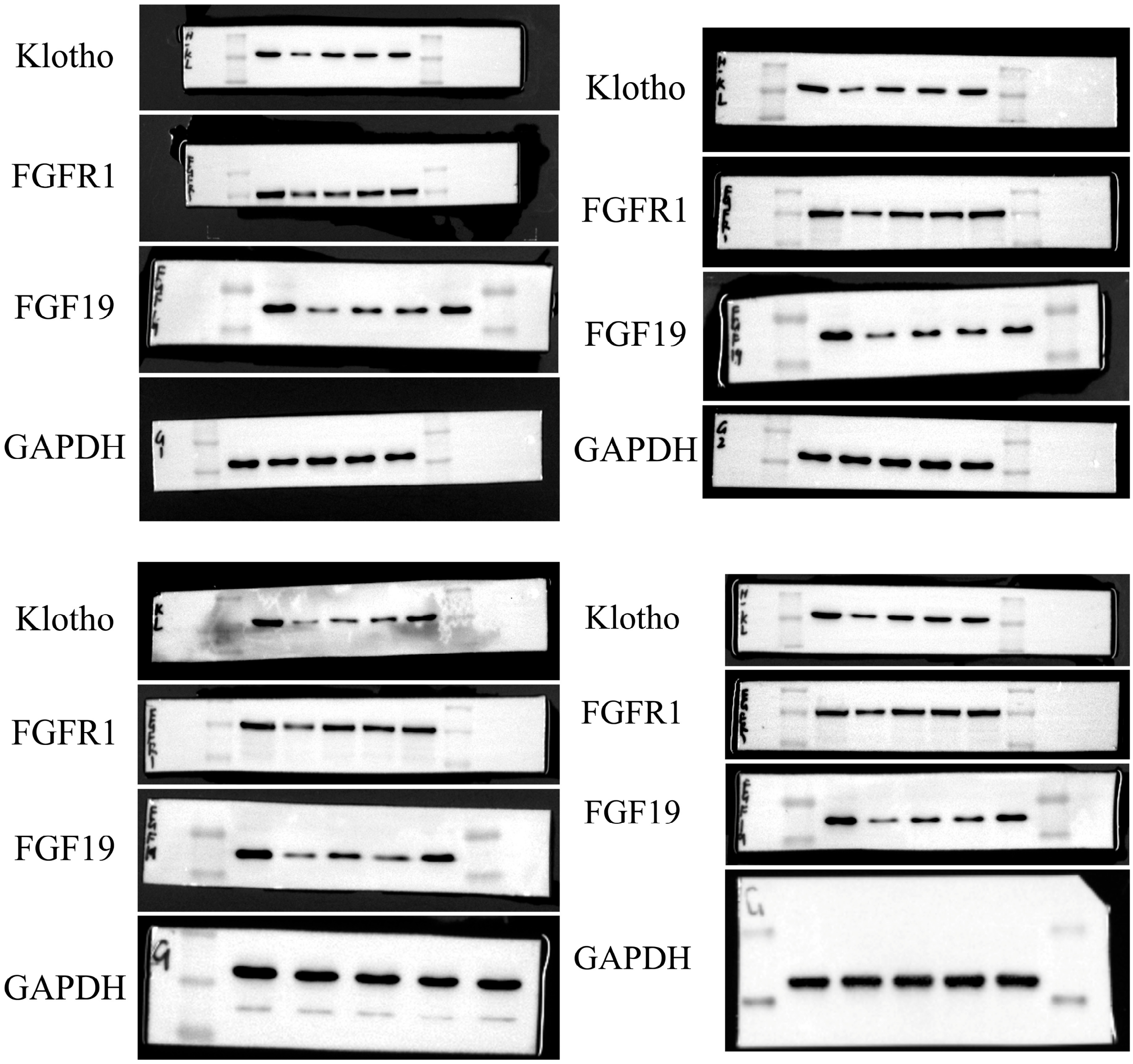

Figure 3B

Original gel images corresponding to Figure 4B

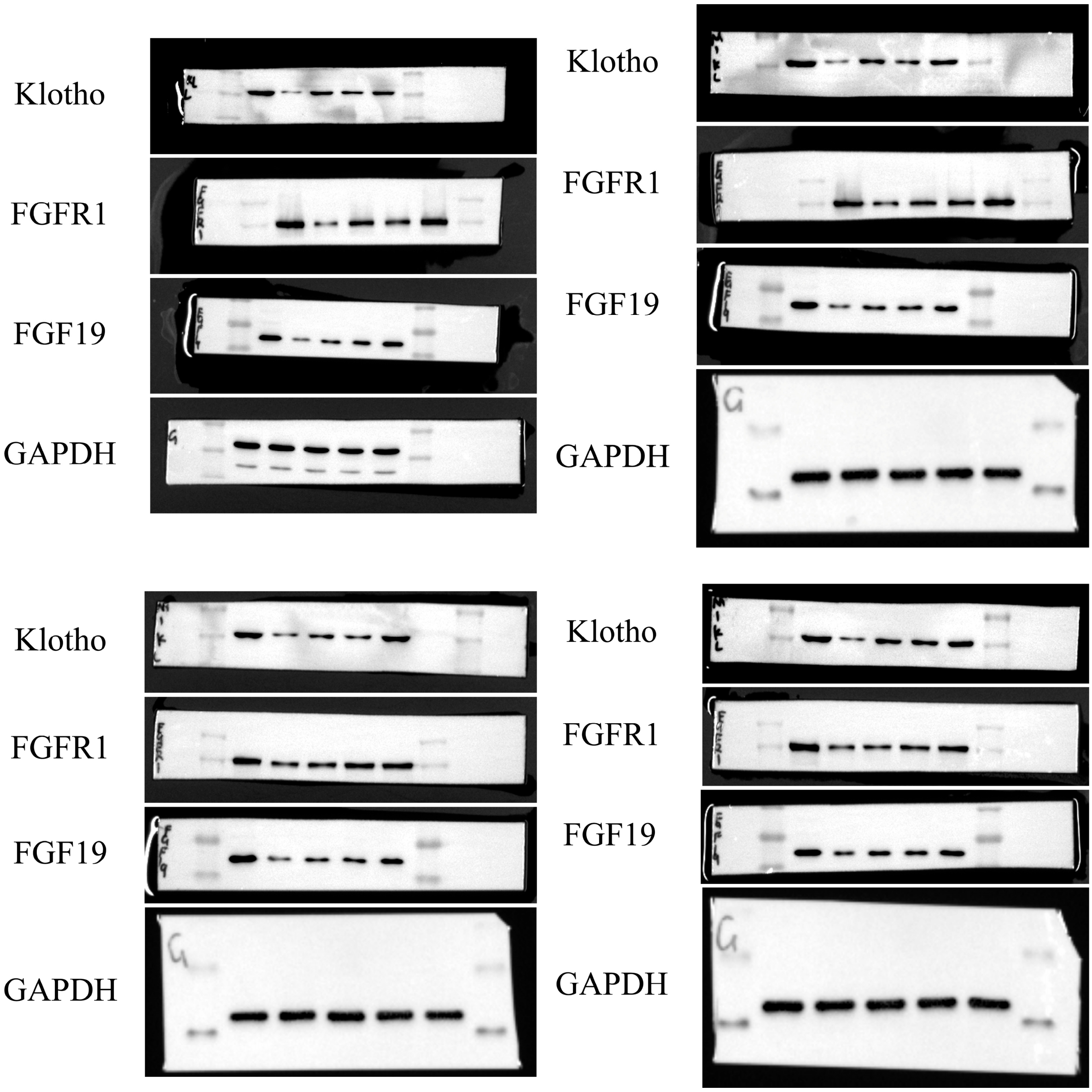

Figure 4B

Original gel images corresponding to Figure 7D

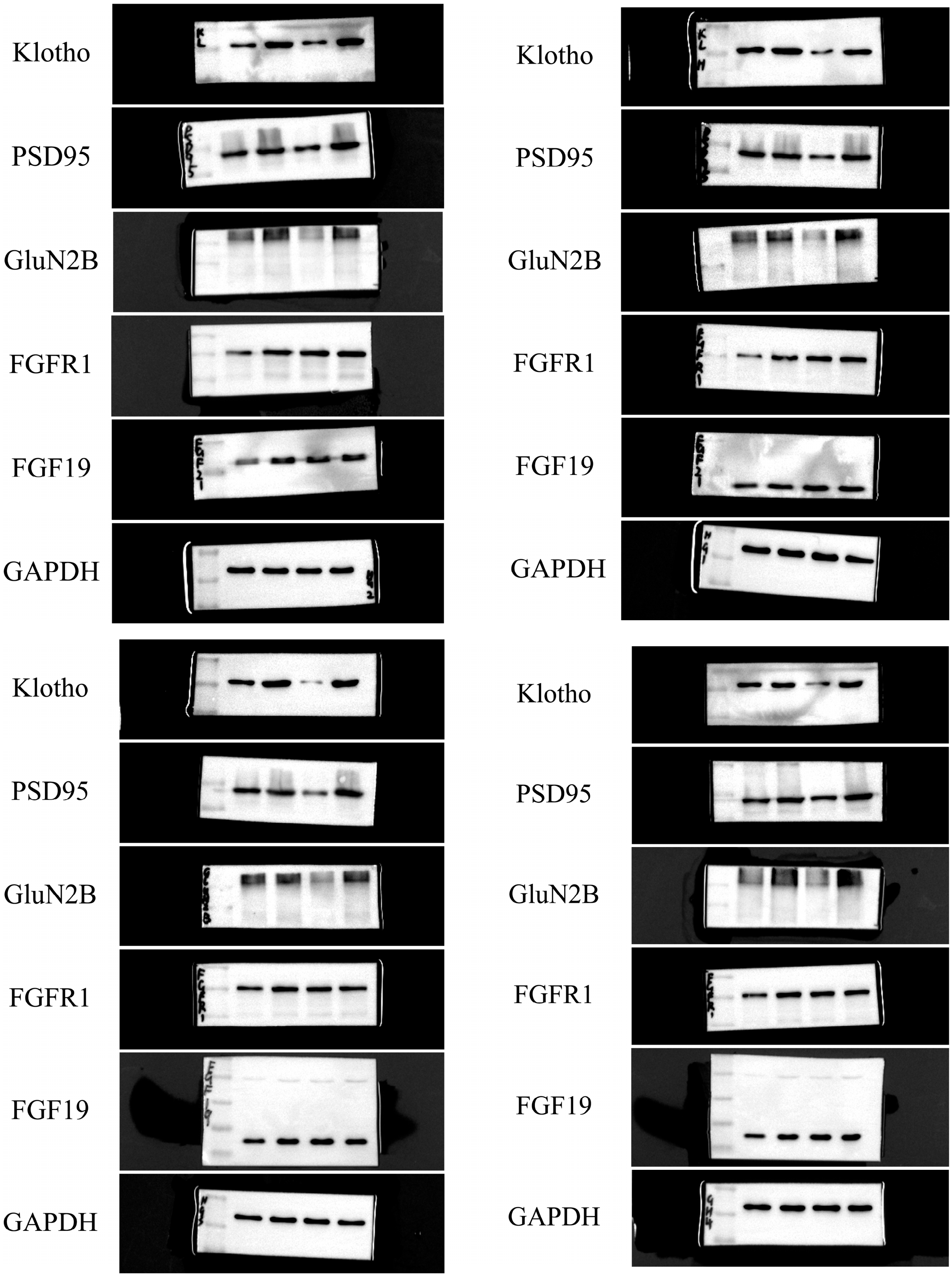

Figure 7D

Original gel images corresponding to Figure 7E

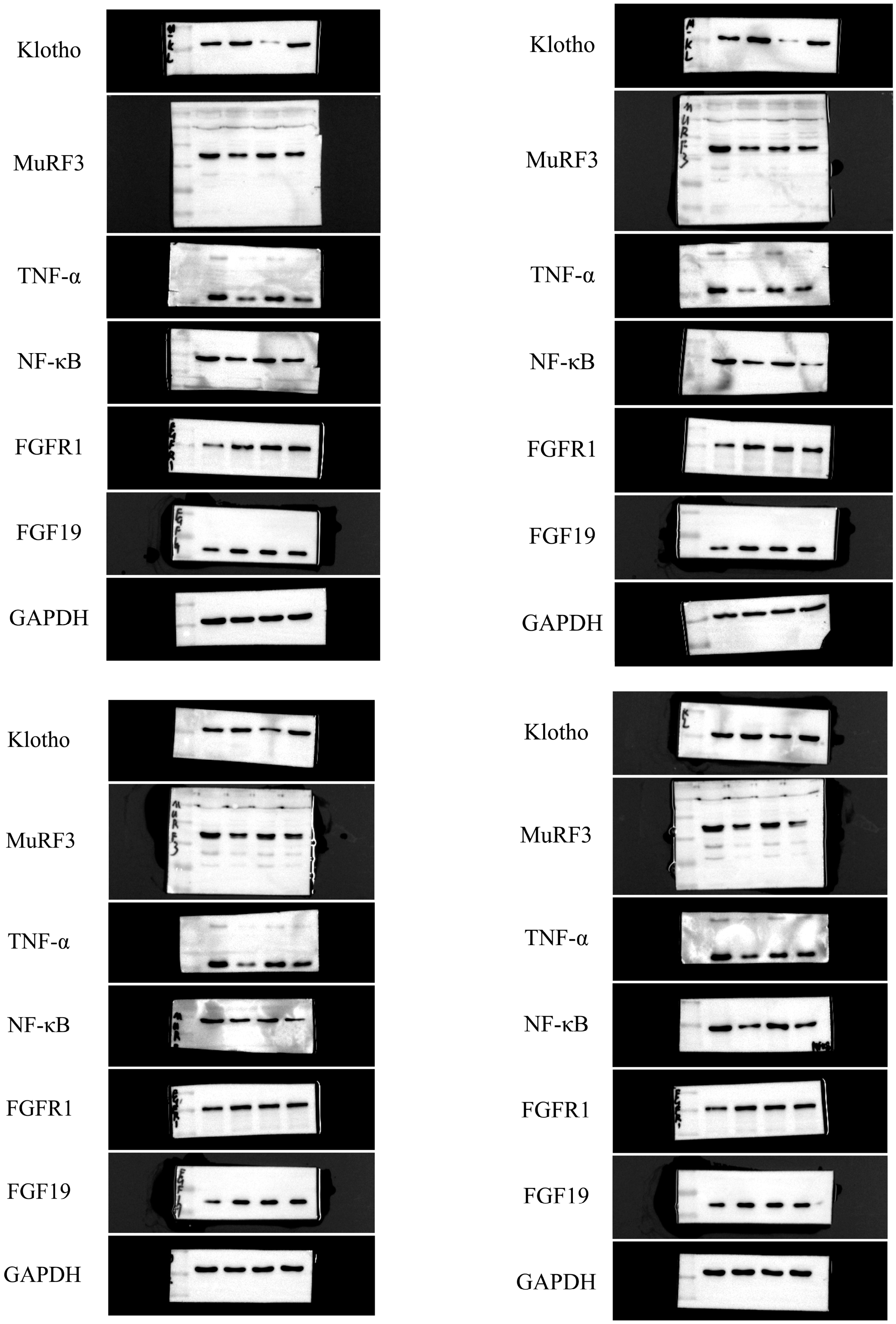

Figure 7E
